# Female Rats Are Resistant to Cognitive, Motor and Dopaminergic Deficits in the Reserpine-Induced Progressive Model of Parkinson’s Disease

**DOI:** 10.3389/fnagi.2021.757714

**Published:** 2021-10-25

**Authors:** Alvaro C. Lima, Ywlliane S. R. Meurer, Vinicius S. Bioni, Débora M. G. Cunha, Narriman Gonçalves, Leonardo B. Lopes-Silva, Marcela Becegato, Manuela B. L. Soares, Gabriela F. Marinho, José R. Santos, Regina H. Silva

**Affiliations:** ^1^Behavioral Neuroscience Laboratory, Department of Pharmacology, Universidade Federal de São Paulo, São Paulo, Brazil; ^2^Memory and Cognition Studies Laboratory, Department of Psychology, Universidade Federal da Paraíba, João Pessoa, Brazil; ^3^Behavioral and Evolutionary Neurobiology Laboratory, Department of Biosciences, Universidade Federal de Sergipe, Itabaiana, Brazil

**Keywords:** parkinsonism, motor impairment, cognitive deficits, sexual dimorphism, tyrosine hydroxylase, estrogen

## Abstract

Parkinson’s disease (PD) is the second most common neurodegenerative disease. The main symptoms are motor signs such as resting tremor and difficulty in initializing movements. Non-motor alterations, such as cognitive deficits, can precede the motor symptoms. PD is more frequent in men than women. The mechanisms related to this difference are not completely understood. There is evidence that females present distinct characteristics in dopaminergic function compared to males. While the severity of motor impairments is often compared between sexes, little is known about sex differences in the prodromal stage. Most animal models of PD present acute severe motor impairment, which precludes the study of non-motor symptoms. Our research group have proposed an adaptation of the classic reserpine protocol, using low doses in a chronic treatment. This method allows the observation of progressive motor impairment as well as premotor deficits. Here we investigate possible behavioral and neuronal sex differences in the effects of the repeated treatment with a low dose of reserpine in rats. Male and female Wistar rats received 10–15 injections of reserpine (0.1 mg/kg) or vehicle, on alternate days. We followed-up the estrous cycle phases and conducted motor and cognitive assessments (catalepsy, open field, oral movements and object recognition tests). The euthanasia occurred 48 h after the 10th or 15th injections, with the collection of blood for the quantification of sex hormones and brains for tyrosine hydroxylase (TH) immunohistochemistry in the substantia nigra pars compact (SNpc). Reserpine induced progressive catalepsy, involuntary oral movements and cognitive deficits in male rats. The behavioral effects of reserpine were attenuated (motor) or absent (cognitive) in females. Reserpine decreased TH immunoreactivity in males, but not in females. Estrogen levels in females negatively correlated with catalepsy duration. Our findings show that females present a delay and/or a prevention in the reserpine-induced motor alterations in the progressive PD model, compatible with the lower prevalence of this disease in women. Further, females were protected from the deficit in object recognition at the prodromal stage. The absence of reserpine-induce decrease in TH immunoreactivity suggests that differences in dopaminergic function/plasticity are related to this protection in female sex.

## Introduction

Parkinson’s disease (PD) is the most common motor disorder and the second most common age-related neurodegenerative disease (Tysnes and Storstein, 2017). The disease is mainly characterized by motor features such as resting tremor, bradykinesia, rigidity, and postural instability (Moustafa et al., 2016), which are associated with loss of dopaminergic nigrostriatal neurons (Alexander, 2004).

PD also includes non-motor symptoms, such as autonomic dysfunctions, cognitive abnormalities, psychiatric symptoms (anxiety, depression), and sleep disorders, some of which may precede clinical diagnoses (Brown and Marsden, 1990; Chaudhuri and Schapira, 2009). Cognitive impairments are frequently reported in PD patients, particularly working and spatial memory deterioration (Gillies et al., 2004), attentional deficits (Benedetti et al., 2001), and recognition memory impairment (Wirdefeldt et al., 2011). Moreover, the cognitive impairments may be among the earliest symptoms in PD patients (Chaudhuri and Naidu, 2008).

The incidence of PD is 1.5–2.0 times higher in men than in women (Van Den Eeden et al., 2003; de Lau and Breteler, 2006; Wirdefeldt et al., 2011; Tysnes and Storstein, 2017). Furthermore, there is a delay in the symptoms onset in females, which is possibly related to a neuroprotective effect of estrogen on the nigrostriatal dopaminergic system (Gillies et al., 2004) or to sex differences in the physiology of dopaminergic neurotransmission (Haaxma et al., 2007). Clinical studies have demonstrated that the lowest severity of motor symptoms of PD in women was correlated with a higher period of estrogen exposure along lifetime. In addition, hormonal decrease or deprivation increases the risk of developing PD (Benedetti et al., 2001; Haaxma et al., 2007).

On the other hand, sex differences in prevalence and prognosis of non-motor symptoms in PD remain controversial. Non-motor symptoms can often reduce quality of life even more significantly than motor alterations (Todorova et al., 2014). Previous studies reported that women show higher predisposition to develop fatigue, constipation, pain, and depression, whereas male PD patients present more serious cognitive deficits (Scott et al., 2000; Solimeo, 2008; Martinez-Martin et al., 2012).

The mechanisms underlying sexual differences in PD, particularly regarding non-motor symptoms, have received little attention. In this regard, some authors pointed out that estrogen is neuroprotective. The protective effect of estradiol might be mediated by a suppressive effect on the dopamine transporter (DAT) function (Watson et al., 2006; McArthur et al., 2007). However, whether or not estradiol protects the dopaminergic pathways in experimental PD appears to vary according some experimental conditions (e.g., neurotoxin used, the severity of the lesion, the strain and species of rodent and the treatment regimen) as well as sex (McArthur et al., 2007). For example, in a previous study Bispo et al. (2019) have shown that ovariectomized females have decreased catalepsy compared to intact male and female rats, although showing a loss in TH + cells in SNpc.

The repeated low-dose reserpine protocol has been used as a model of progressive parkinsonism, showing phenomenological (progressive motor alterations) and construct (reduced dopaminergic parameters, increased oxidative stress, and augmented alpha-synuclein expression) similarities with PD (Fernandes et al., 2012; Santos et al., 2013; Leão et al., 2015, 2017, 2021; Ikram and Haleem, 2019). Unlike neurotoxin models, which lead to acute severe motor impairment, the low-dose repeated reserpine protocol allows the observation of progressive motor impairments as well as premotor deficits such as cognitive prejudice, anxiety and nociception alterations (Santos et al., 2013; Leal et al., 2019; Cintra et al., 2021; Dos Santos et al., 2021). This is of relevance when studying PD sex differences because women seem to have a later onset of the motor symptoms, while non-motor prodromic features are poorly understood. Thus, the present study aimed to evaluate sex differences on motor, cognitive, and neurochemical alterations in rats submitted to the low-dose reserpine-induced progressive model of PD.

## Materials and Methods

### Animals

Female (*n* = 40) and male (*n* = 20) Wistar rats (7–8 months old) were used in this study. All animals were housed in groups of 4 per cage (30 cm × 42 cm × 16 cm) under controlled airflow, acoustic isolation, and temperature at 22 ± 1°C with a 12 h light/12 h dark cycle (light on at 7:30 a.m.). All animals had free access to water and food. Animals used in this study were handled according to the Brazilian law for the use of animals in research (Law Number 11,794) and all procedures were approved by the local ethics committee (protocol number 3683250517). All efforts were made to minimize animal pain, suffering or discomfort and to reduce the number of animals used.

### Drug Treatment and General Procedures

**FIGURE 1:**
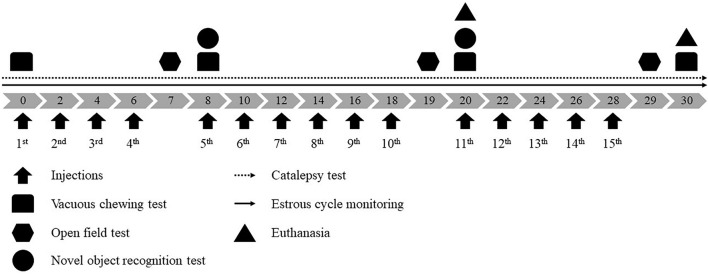
Experimental design. Gray arrows indicate days of treatment and black arrows pointed up indicate vehicle or 0.1 mg/kg reserpine s.c. injections.

Reserpine (Res, Sigma Chemical Co., United States) was dissolved in glacial acetic acid (1%) and then diluted to the correct concentration with distilled water. The vehicle solution (Veh) consisted of the same amount of acetic acid and water as in the reserpine solution. Rats were randomly assigned to one of four groups: Female-Veh (*n* = 20), Male-Veh (*n* = 10), Female-Res (*n* = 20), and Male-Res (*n* = 10). Animals received subcutaneous injections of vehicle (Veh) or 0.1 mg/kg of reserpine (Res) at a volume of 1 mL/kg body weight, every 48 h for 20 or 30 days of treatment, completing 10 or 15 injections, respectively. Animals were euthanized 48 h after the 10th or 15th injections (Figure 1).

Across treatment, animals were submitted to the following procedures: (1) estrous cycle monitoring (daily); (2) catalepsy test (before the first injection and daily across treatment); (3) assessment of oral movements before the first injection and 48 h after the 4th, 10th, and 15th injections; (4) evaluation of open field behavior 24 h after the 4th, 10th, and 15th injection; (5) novel object recognition test after the 4th and 10th. If the procedure was held in a treatment day, it was conducted before the injection (from 12:00 a.m. to 14:00 p.m.). Experimental design is shown in Figure 1.

In order to evaluate the estrous cycle, vaginal smears were collected by vaginal lavage using saline at room temperature. The vaginal smears collected were placed immediately on the glass slide, colored with methylene blue and analyzed under the optical microscope in 10× magnification, following the methods described by Marcondes et al. (2002).

### Behavioral Tests

#### Catalepsy Test

The catalepsy behavior was assessed by placing the animal’s forepaws on a horizontal bar positioned 9 cm above the bench surface while the hind paws rested on the bench. The animals were free to move after they were placed in this position by the experimenter. Catalepsy was defined as an immobile posture (keeping both forepaws on the bar, see Supplementary Video 1). The latency to withdraw one or both forepaws from the bar was measured up to a maximum of 180 s (Sanberg et al., 1988). Three trials for animal in each observation day were carried out and the results were analyzed considering the mean value of these trials. In addition to the daily analysis of catalepsy curve, analyzes were performed in phases, considering means of 5 observations: basal (without injection), prodromal phase (1st∼5th), early motor phase (6th∼10th) and late motor phase (11th∼15th).

#### Vacuous Chewing Test

The animals were individually placed in a wire cage (20 × 20 × 15 cm). Mirrors were positioned under and behind it to allow behavioral quantification when animal faced away from the observer. The number of vacuous chewing movements (mouth openings in the vertical plane not directed toward physical material, see Supplementary Video 2) was measured continuously for 10 min.

#### Open Field Test

The animals were submitted to the open field in order to evaluate spontaneous locomotor activity. The apparatus was a circular arena (67 cm in diameter) surrounded by a 40 cm high wall, made of wood and painted black. Animals were placed in the center of the apparatus for free exploration for 5 min. Distance traveled in the whole arena (in meters) was evaluated. The sessions were recorded by a digital camera and the behavioral parameters were registered by Any-maze^®^ (Stoelting. Co., IL, United States).

#### Novel Object Recognition Test

The novel object recognition (NOR) task was carried out in the same arena used in the open field test. In each day of test two pairs of objects were used randomly among animals. The objects used were all made of plastic material and filled with cement to ensure that animals could not displace them. The objects differed in height, width, color and shape. A previous experiment demonstrated that rats do not show spontaneous preference for any of these objects. In the training session, the animals were exposed to two copies of an object for 5 min. The same procedure was carried out 1 h later (test session), except that one of the objects was replaced for a new one. Different sets of objects were used on the 20th day (10th injection) to avoid a possible recognition of objects used on the 8th day (4th injections). The apparatus and objects were cleaned with a 5% alcohol solution after each behavioral session. The sessions were recorded by a digital camera and the behavioral parameters were registered by Any-maze^®^. The time spent exploring each object was measured in both sessions. Exploration behavior was defined by touching with forepaws or nose, sniffing and biting the objects. Only animals that showed total object exploration greater than 3 s during the training were included in the analysis. The discrimination index was calculated as exploration difference (old – new object)/total objects exploration (old + new object).

### Tissue Processing and Tyrosine Hydroxylase Immunohistochemistry

Upon completion of behavioral procedures, animals were pre-anesthetized with fentanil (0.5 mg/kg, i.p.) and acepran (1.0 mg/kg, i.m.). After 5 min they were deeply anesthetized with an intraperitoneal injection of 9.0 mg/kg ketamine chloridate and xylazine and transcardially perfused with 200–250 mL phosphate-buffered saline (PBS), pH 7.4, containing 0.2% heparin, followed by 200 mL PBS with 4% paraformaldehyde 0.1 M. The brains were removed from the skull, post fixed in the same fixative solution and stored at 4°C. After 24 h, we transferred brains to a solution containing sucrose 30% 0.1 M PBS, at 4°C. Each brain was fixed in Tissue-Tek^®^ (Sakura, Japan) at −20°C. Then, we serially sliced the brains in the coronal plane into 50 μm thick sections with a cryostat microtome (Leica, Germany) at a temperature of −20°C. Following tissue processing, we performed immunohistochemistry for TH, using a free-floating protocol. Sections were washed four times with PBS (pH 7.4) for 5 min each and consecutively washed with 0.03% H_2_O_2_ solution for 20 min to reduce endogenous peroxidase activity. For detection of TH, sections were incubated with rabbit anti-tyrosine hydroxylase polyclonal antibody (cat # AB152 Chemicon, United States, 1:5,000) diluted in triton x-100 0.4% and PBS with 2% albumin serum, for 18–24 h at room temperature. Afterward, sections were incubated with goat biotinylated anti-rabbit IgG (Vector Labs, United States, 1:1,000) diluted with triton x-100 0.4% NaCl and PBS for 2 h at room temperature, followed by washing steps, and incubated with avidin-biotin-peroxidase solution (ABC Elite kit, Vector Labs, Burlingame, United States) for another 2 h. The reaction was developed by adding of 3,3-diaminobenzidine (DAB—Sigma-Aldrich, United States) and 0.01% H_2_O_2_ 0.1 M phosphate buffer solution for 3 min. Then, we left sections to dry, dehydrated in a graded alcohol series, cleared in xylene, and coverslipped with Entellan (Merck). All sections were immunostained concomitantly, to minimize possible background differences between samples. Sections were examined under brightfield illumination with an optical microscope (Nikon Eclipse 80i), attached with a motorized stage (MBF Bioscience) and CCD camera (Nikon, DXM-1200) to record images. In order to estimate the number of TH + cells in SNpc, we analyzed 8–12 sections of each animal, equally distributed at the rostral, medium and caudal levels of the structure. All TH + cells of SNpc on each section were registered. The mean of all measures was calculated, and the data were normalized by the respective control.

### Estrogen Dosage

During the transcardiac perfusion, the blood was collected with a coagulation activator and separator gel. Posteriorly, it was centrifuged at 5,000 rpm for 10 min at 4°C, the pliers were stored at −80°C until the assays. The determination of estradiol concentrations in plasma was performing using electroimmunoluminescence method (Tiskievicz et al., 2011) in the Hermes Perdini Analysis Laboratory.

### Data Analysis

Data normality and homogeneity of variances were tested by the Shapiro-Wilk and Levene’s tests, respectively. Behavioral data was grouped in two sets of analysis: animals that received 10 and 15 injections. The average of 2 days of catalepsy behavior, catalepsy phases, oral movements and locomotion in the open field were analyzed by two-way repeated-measure ANOVA followed by Sidak’s *post hoc* test. Discrimination indexes in NOR and percentage of occurrence of estrous cycle phases were analyzed by two-way ANOVA followed by Sidak’s *post hoc* test. Non-parametric data from TH + quantification and the hormonal levels were analyzed by the Mann-Whitney *U*-test in each group separately. In these cases, Bonferroni correction was carried out and alpha value was set at 0.025. Spearman’s rank correlation coefficient was used to investigate a possible association between hormonal levels and motor performance in the catalepsy test. Correlations in vehicle and reserpine groups were investigated separately, considering the different treatment durations. Results were expressed as mean ± SEM and *p* < 0.05 was considered to reflect significant differences.

## Results

### Catalepsy Test

**FIGURE 2:**
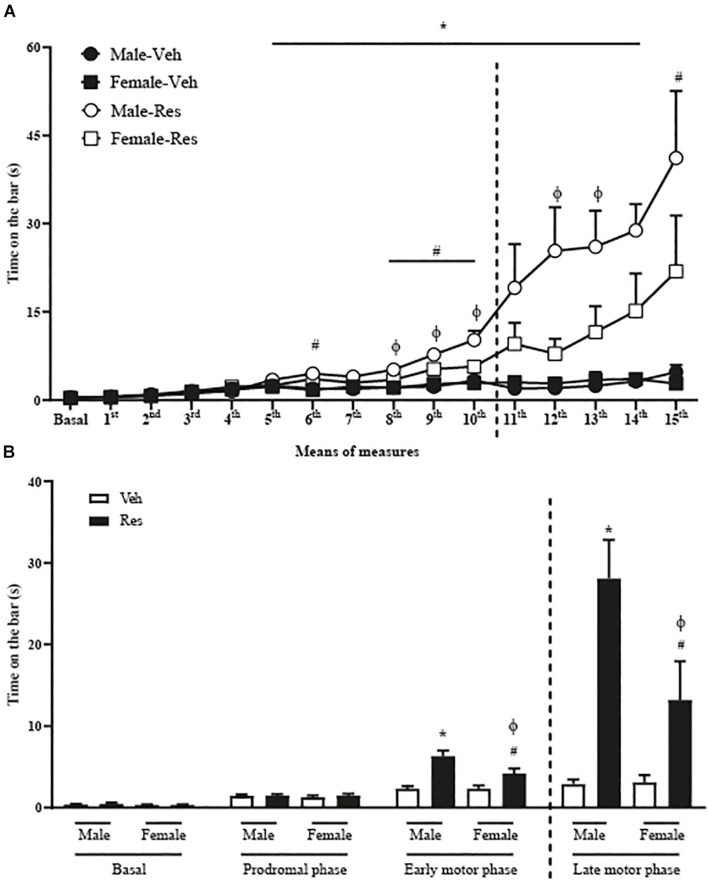
Effects of repeated treatment with 0.1 mg/kg reserpine (Res) or vehicle (Veh) on catalepsy behavior in female and male rats throughout the treatment **(A)** and grouped in phases **(B)** basal (without injection of Res or Veh), prodromal (1∼5 injections), early motor (6∼10 injections), and late motor (10∼15 injections). Dashed lines indicate the euthanasia of half the animals. *p < 0.05 comparing Male-Res and Male-Veh, #*p* < 0.05 comparing Female-Res and Female-Veh and ϕ*p* < 0.05 comparing Male-Res and Female-Res (Two-way repeated-measure ANOVA followed by Sidak’s *post hoc* test). Values were expressed by mean ± SEM. Individual data are displayed in Supplementary Tables 1, 2).

A two-way repeated-measure ANOVA for all animals until the 10th injection of Veh or Res revealed significant effects of time [*F*_(10, 560)_ = 61.014; *p* < 0.001], treatment (Veh and Res) [*F*_(1, 56)_ = 16.740; *p* < 0.001], and interactions between time and treatment [*F*_(10, 560)_ = 16.962; *p* < 0.001], time and sex (Male and Female) [*F*_(10, 560)_ = 3.278; *p* = 0.021] and time, treatment and sex [*F*_(10, 560)_ = 3.307; *p* = 0.021]. Another two-way repeated-measure ANOVA (only with animals that received 15 injections of Veh or Res) revealed significant effects of time [*F*_(15, 390)_ = 15.918; *p* < 0.001], treatment [*F*_(1, 26)_ = 22.356; *p* < 0.001], sex [*F*_(1, 26)_ = 4.446; *p* = 0.045] and interactions between time and treatment [*F*_(15, 390)_ = 10.951; *p* < 0.001] and sex and treatment [*F*_(1, 26)_ = 4.605; *p* = 0.041]. The Sidak’s *post hoc* test revealed significant increases in the duration of catalepsy behavior in Male-Res group compared to Male-Veh from the 6th to the 15th measures. Female-Res group exhibited significant increases in the duration of catalepsy compared to Female-Veh in the 6th, 8th, 9th, 10th, and 15th measures. In addition, male-Res showed significant increases in the time on the bar compared to Female-Res on the 8th, 9th, 10th, 12th, and 13th measures (Figure 2A).

We also analyzed the catalepsy progression in means of 5 injections in basal (without injections of Veh or Res), prodromal (1st∼5th injections), early motor (6th∼10th injections) and late motor (11th∼15th injections) phases. Two-way repeated-measure ANOVA (considering all animals until the 10th injection of Veh or Res) revealed significant effects of phases (basal, prodromal and early motor phase) [*F*_(2, 112)_ = 122.805; *p* < 0.001], treatment (Veh or Res) [*F*_(1, 56)_ = 17.028; *p* < 0.001], and interactions between time and treatment [*F*_(2, 112)_ = 27.013; *p* < 0.001] and time, treatment and sex [*F*_(2, 112)_ = 4.139; *p* = 0.039].

The analysis of the phases of catalepsy by two-way repeated-measure ANOVA (considering only animals that received 15 injections) showed significant effects of phases [*F*_(3, 78)_ = 31.261; *p* < 0.001], treatment [*F*_(1, 26)_ = 22.362; *p* < 0.001], sex [*F*_(1, 26)_ = 4.556; *p* = 0.042], and interactions between phases and treatment [*F*_(3, 78)_ = 20.288; *p* < 0.001] and sex and treatment [*F*_(1, 26)_ = 4.635; *p* = 0.041]. The Sidak’s *post hoc* test revealed that Male-Res show increased time in catalepsy duration compared to Male-Veh and Female-Res on early and late motor phases (Figure 2B).

### Vacuous Chewing Movements

A two-way repeated-measure ANOVA (considering all animals until the10th injection of Veh or Res) revealed significant effects of the interactions between time (basal, 4th and 10th injections) and treatment (Veh or Res) [*F*_(2, 66)_ = 3.807; *p* = 0.027] and treatment and sex [*F*_(1, 33)_ = 5.107; *p* = 0.031].

Another two-way repeated-measure ANOVA (considering only animals that received 15 injections) revealed a significant effect of time [*F*_(3, 42)_ = 3.767; *p* = 0.018] and interaction between time and sex [*F*_(3, 42)_ = 4.274; *p* < 0.010].

**FIGURE 3:**
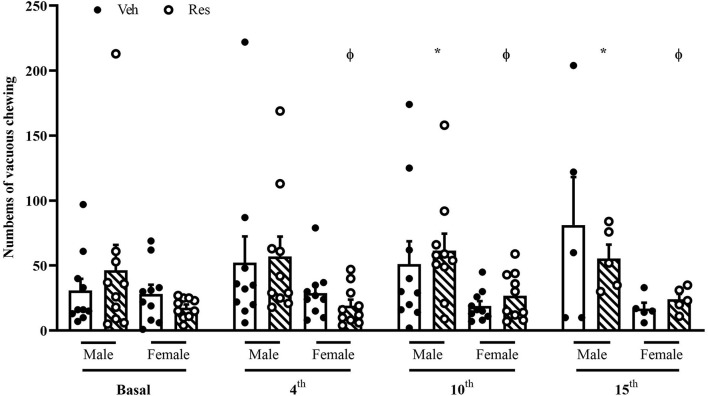
Effects of repeated treatment with 0.1 mg/kg reserpine (Res) or vehicle (Veh) on the vacuous chewing (VCM) behavior in female and male rats. *p < 0.05 comparing Res and Veh and ϕ*p* < 0.05 comparing Male-Res and Female-Res (Two-way repeated-measure ANOVA followed by Sidak’s *post hoc* test). Values were expressed by mean ± SEM. Black and circles represent individual data of Veh and Res animals, respectively.

The Sidak’s *post hoc* test exhibited a significant effect of Res in Male animals in the 3rd and 4th measures (10th and 15th administrations, respectively). There was also a sex difference from the 2nd until the last measure (4th, 10th, and 15th injections). Reserpine-treated females did not show an increase in the vacuous chewing (Figure 3).

### Open Field Test

A two-way repeated-measure ANOVA (considering all animals that went through OF 48 h after the 4th and 10th injections of Veh or Res) revealed significant effects of time [*F*_(1, 36)_ = 47.821; *p* < 0.001], treatment [*F*_(1, 36)_ = 6.378; *p* = 0.016], sex [*F*_(1, 36)_ = 17.190; *p* < 0.001] and interaction between time and treatment [*F*_(1, 36)_ = 9.571; *p* = 0.004].

Another two-way repeated-measure ANOVA (considering only animals that went through OF 48 h after the 4th, 10th, and 15th injections) exhibited significant effects of time [*F*_(2, 32)_ = 64.913; *p* < 0.001], treatment [*F*_(1, 16)_ = 22.256; *p* < 0.001], sex [*F*_(1, 16)_ = 9.475; *p* = 0.007], and interaction between time and treatment [*F*_(2, 32)_ = 34.727; *p* < 0.001] and time, treatment and sex [*F*_(2, 32)_ = 8.018; *P* = 0.002].

**FIGURE 4:**
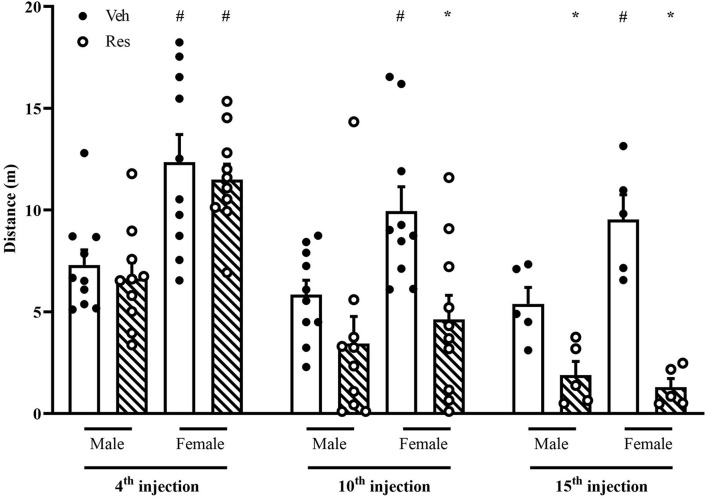
Effects of repeated treatment with 0.1 mg/kg reserpine (Res) or vehicle (Veh) on locomotion in the open field in female and male rats. *p < 0.05 comparing RES and Veh. ^#^*p* < 0.05 comparing males and females with the same treatment (Two-way repeated-measure ANOVA followed by Sidak’s *post hoc* test). Values were expressed by mean ± SEM. Black and circles represent individual data of Veh and Res animals, respectively.

The Sidak’s *post hoc* test revealed a significant increase of locomotion of Female-Veh in the three measures. In addition, 48 h after the 10th and 15th injections, the Sidak *post hoc* test showed Res decreased locomotion in both sexes (Figure 4).

### Novel Object Recognition

**FIGURE 5:**
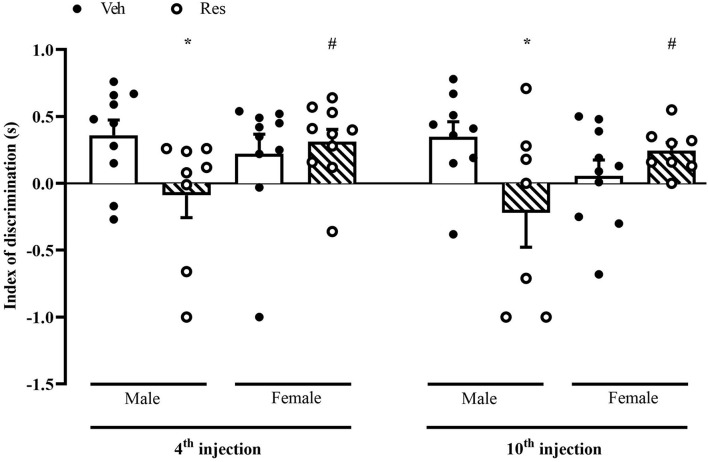
Effects of repeated treatment with of 0.1 mg/kg reserpine (Res) or vehicle (Veh) on object recognition memory in female and male rats. *p < 0.05 comparing Res and Veh ^#^*p* < 0.05 comparing Male-Res and Female-Res (Two-way ANOVA followed by Sidak’s *post hoc* test). Values were expressed by mean ± SEM. Black and circles represent individual data of Veh and Res animals, respectively.

The NOR analysis included only animals that obtained a total exploration time greater than 3 s in the training phase. A two-way ANOVA for the discrimination index considering the first and second measures (48 h after the 4th and 10th injections of Veh or Res) exhibited a significant effect of the interaction between treatment and sex in the first (after the 4th injection of Veh or Res) [*F*_(1, 34)_ = 4.302; *p* = 0.046] and second (after the 10th injection of Veh or Res) [*F*_(1, 30)_ = 6.947; *p* = 0.013] measures. The Sidak’s *post hoc* test revealed significant effects of Res in Male in both tests. On the other hand, females did not show the same effect, and differed from males treated with Res (Figure 5).

### Tyrosine Hydroxylase Immunohistochemistry

**FIGURE 6:**
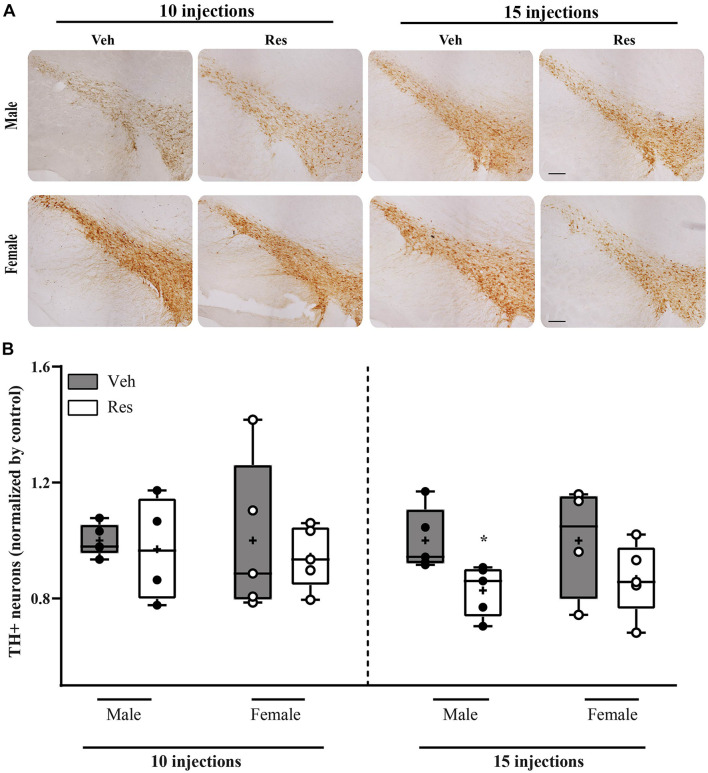
Effects of repeated treatment with 0.1 mg/kg reserpine (Res) or vehicle (Veh) on the number of TH immunoreactive cells in substantia nigra pars compacta in female and male rats after 10 or 15 injections. **(A)** Representative photomicrographs of brain coronal sections of substantia nigra pars compacta. Scale bar: 100 μm. **(B)** Quantification of TH immunoreactive cells. Horizontal lines in the middle of each box indicates the median TH levels, while the top and bottom borders of the box mark the 25th and 75th percentiles, respectively. Black and circles represent individual data of Veh and Res animals, respectively. *p < 0.05 comparing Res and Veh (Mann-Whitney *U*-test). Data of individual brain sections are displayed in Supplementary Table 3.

Mann-Whitney *U*-test did not show differences in TH + levels between vehicle and reserpine for females and males after ten injections of Res (*U* = 13.000; *p* = 0.917, and *U* = 9.000; *p* = 0.905, respectively). On the other hand, fifteen injections of reserpine reduced TH expression of Male-Res when compared to Male-Veh (*U* = 0.000; *p* = 0.008). Female-Res did not show differences in TH expression compared with Female-Veh (*U* = 5.000; *p* = 0.286) (Figure 6).

### Evaluation of Estrous Cycle and Estradiol Levels

**FIGURE 7:**
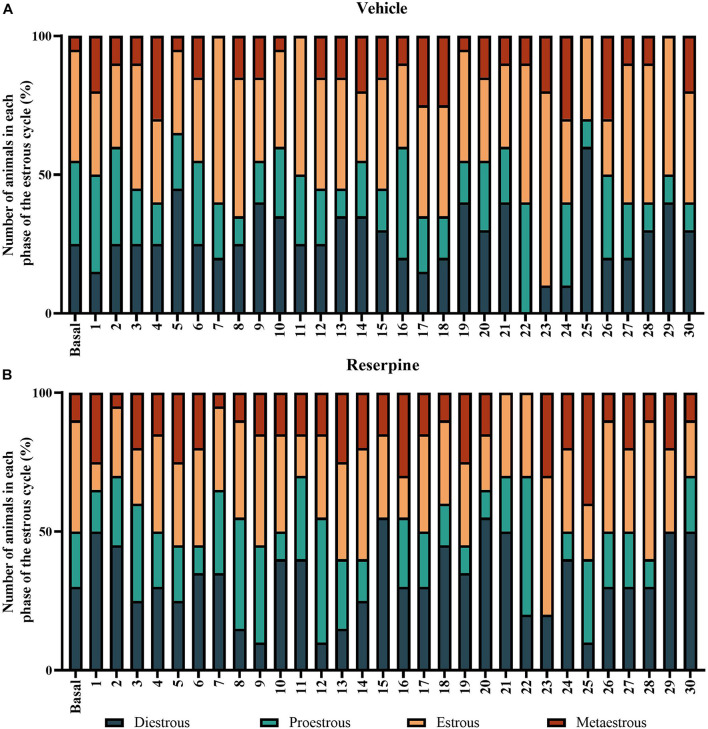
Prevalence of the phases of estrous cycle throughout the 30-day repeated treatment with vehicle **(A)** or 0.1 mg/kg reserpine **(B)** in female rats. Values were expressed by percentage of animals in each phase in each day of the protocol.

In order to verify the influence of the estrous cycle throughout the treatment, we monitored the estrous cycle phases throughout the protocol. The prevalence of each cycle phase in each day of the protocol was determined for each female group. A two-way ANOVA applied to the percentage of occurrence of the cycle stage between treatments showed only a significant effect of cycle phases [*F*_(3, 240)_ = 42.194; *p* < 0.001], probably because the duration of each phase is physiologically different. There was a lower overall occurrence of the metaoestrous phase (Table 1 and Figure 7). There were no differences regarding treatment.

**TABLE 1:** Percentage of occurrence of each phase of the estrous cycle during treatment with 0.1 mg/kg reserpine (Res) or vehicle (Veh).

	**Veh**	**Res**
Diestrous	27.10 ± 2.12	32.58 ± 2.41
Proestrous	20.64 ± 1.75	20.48 ± 2.22
Estrous	38.87 ± 1.98	30.48 ± 1.68
Metaestrous	13.39 ± 1.60	16.45 ± 1.59

*Values are expressed in means of percentage of animals in each phase across treatment ± SEM. (Two-way ANOVA revealed only effect of phases).*

**FIGURE 8:**
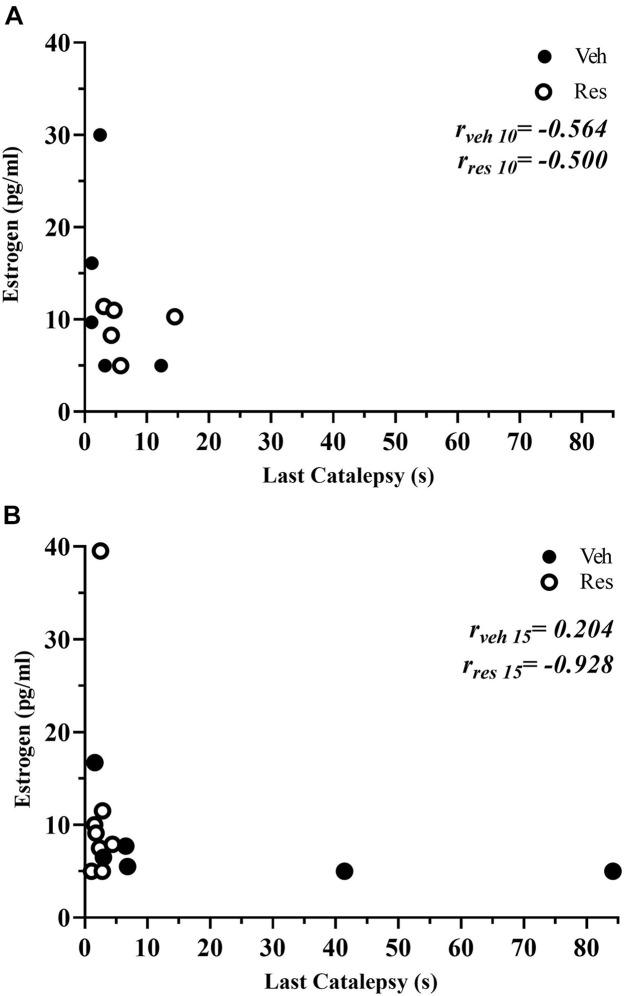
Scatterplots depicting the correlations between the serum estradiol levels and duration of catalepsy of vehicle and reserpine groups that received 10 **(A)** and 15 injections **(B)**.

A non-parametric analysis (Mann-Whitney test) did not show differences in estrogen levels in vehicle and reserpine females after 10 or 15 injections (*U* = 12,000; *p* = 0.0916, and *U* = 16.000; *p* = 0.296, respectively, Table 2). A Spearman’s rank-order correlation was run to investigate a possible association between estradiol levels and motor performance (last catalepsy measure) for each group at both treatment lengths. There was a strong negative correlation between estradiol and catalepsy immobility for reserpine-treated group at the 15th injection [*r*_s(6)_ = −0.928; *p* = 0.008], but not at the 10th injection [*r*_s(5)_ = −0.500; *p* = 0.391] or vehicle-treated animals at the 10th [*r*_s(5)_ = −0.564; *p* = 0.322] and 15th [*r*_s(8)_ = 0.204; *p* = 0.629) injections (Figure 8).

**TABLE 2:** Serum estradiol levels after 10 or 15 injections of repeated treatment with 0.1 mg/kg reserpine (Res) or vehicle (Veh) in female Wistar rats.

	**Veh**	**Res**
10 injections	13.16 ± 4.68	9.20 ± 1.05
15 injections	11.94 ± 4.02	7.73 ± 1.84

*Data are expressed as mean of estradiol levels (pg/mL) and SEM (Mann-Whitney test).*

## Discussion

In this work, we investigated sex differences in motor, cognitive and neurochemical alterations at different stages of repeated low-dose reserpine administration, a progressive animal model of PD (Santos et al., 2013; Leão et al., 2015, 2017; Peres et al., 2016; Campelo et al., 2017; Lins et al., 2018; Bispo et al., 2019).

Corroborating previous studies, we observed that reserpine treatment induced a progressive motor impairment in male rats (Fernandes et al., 2012; Santos et al., 2013; Leão et al., 2017; Lins et al., 2018; Bispo et al., 2019). Interestingly, male rats showed more susceptibility to these motor alterations than female rats. Indeed, our results showed that males had a greater increase in catalepsy duration and vacuous chewing movements when compared to females. Conversely, the decrement in open field spontaneous activity was similar for both sexes. Further, we observed a sex difference in the short-term memory task: only RES-treated male rats showed a reduction in the discrimination index, which was observed prior to the motor deficits onset, in the prodromal phase, and remained throughout the protocol. Moreover, the low-dose repeated reserpine administration caused a decrease in TH immunoreactivity in SNpc only in males. Decreased estrogen levels correlated with increased immobility in the catalepsy test. Overall, we showed sex differences in the development of repeated reserpine-induced parkinsonism, highlighting alterations that occur in non-motor and motor phases, which are poorly understood in terms of sex differences in PD.

Studies have investigated sex differences in the 6-OHDA (Cass et al., 2005; Field et al., 2006; Zarate et al., 2021) and MPTP (Ookubo et al., 2009; Antzoulatos et al., 2010) animal models of PD. However, these protocols promote acute brain injury and rapid instauration of severe motor impairment, which hinders the evaluation of differences in motor deficits progressiveness and non-motor assessments (Saunders-Pullman et al., 1999; Tsang et al., 2000; Datla et al., 2003). Our results showed that the low-dose reserpine injections induced a progressive motor deficit in both male and female rats. Our findings revealed a greater motor impairment in male rats, corroborating previous findings in which catalepsy onset occurred 48 h after 6–8 reserpine injections (Fernandes et al., 2012; Santos et al., 2013; Campelo et al., 2017; Leão et al., 2017). On the other hand, Res-treated females showed a delayed onset of motor deficits. This outcome corroborates a previous study conducted with male and female mice (Leão et al., 2015).

Besides the lower susceptibility to increased catalepsy, female rats were not susceptible to the oral dyskinesia (evaluated by the vacuous chewing movements) induced by repeated treatment with a low dose of reserpine, which was present in males. Previous studies shown increased VCM response in males with the same protocol used here (Fernandes et al., 2012; Peres et al., 2016; Leão et al., 2017). However, one prior study reported increased reserpine-induced VCM response in female mice (Silva et al., 2002) after an acute injection of 1.0 mg/kg reserpine. In this regard, these studies proposed several hypotheses to explain the mechanisms underlying VCM, including dopamine hypofunction. Notably, reserpine promotes dopamine depletion by VMAT2 inhibition and, possibly modulation of dopamine receptor expression in several brain areas. In addition, reserpine increases dopamine autooxidation and oxidative catabolism by MAO, leading to oxidative stress (Abílio et al., 2003; Fuentes et al., 2007; Reckziegel et al., 2013). Accordingly, oral dyskinesia is also related to oxidative stress and is attenuated by antioxidant compounds (Abílio et al., 2003). In this way, it is possible that female rats would have a greater protection against oxidative stress, since they showed higher antioxidant enzyme activity than that observed in males (Siani et al., 2017; Tenkorang et al., 2018; Varmazyar et al., 2019).

Concerning spontaneous locomotion in the open field, our results did not show a sex difference for locomotor deficits in the Res-treated rats. Despite both female groups (Veh and Res), 48 h after the 4th injection, presented a higher locomotor activity when compared with respective male groups, as reported before (Cronan et al., 1985; Ribeiro et al., 2010), our findings showed that both sexes significantly reduced locomotion throughout the experiment, probably due to habituation. Nevertheless, Res-treated rats, irrespective of sex, presented a significant decrease in motor activity 48 h after the 10th and 15th injections. In this regard, other studies have described similar effects for males (Santos et al., 2013; Leão et al., 2017; Lins et al., 2018; Bispo et al., 2019) and females (Bispo et al., 2019). Although there is a visually more pronounced reduction in locomotion in reserpine females than in males in the second open field session, statistical analysis did not show a significant interaction between time (repeated sessions) and sex. In addition, a comparison between percent of locomotion reduction between groups revealed only an effect of treatment (data not shown). The absence of a protection against Res-induced decrement in spontaneous locomotion in females may occur because this behavior does not depend purely on motor skills. Instead, it encompasses factors such as motivation to explore, anxiety-like behavior and habituation after repeated exposures, among others (Walsh and Cummins, 1976; Choleris et al., 2001).

Regarding non-motor alterations, which often appear before motor symptoms, cognitive deficits are highly prevalent in PD (Roheger et al., 2018). The progressive parkinsonism induced by low dose-reserpine is a valuable tool that enables the evaluation of non-motor impairment, including emotional and cognitive deficits, during early stages of parkinsonism development in rodents (Santos et al., 2013; Campelo et al., 2017; Leal et al., 2019). In a prior study, Santos et al. (2013) reported impaired short-term object recognition memory before motor deficits in male rats (48 h after the 4th injection of reserpine). In a similar way, our results showed that Res-treated male presented memory deficits 48 h after the 4th and 10th injections. Importantly, Res-treated female rats did not show object recognition memory deficits. The present study evaluated only one memory test. Thus, whether this sex difference is applicable for cognitive function as a whole remains to be investigated. Beneficial effects of estrogen on memory could be related to neuronal survival, neurotransmission modulation, increased synaptogenesis, and protection against oxidative stress damage (Behl et al., 1995; Maggi et al., 2004; Shulman, 2007; Matheus et al., 2016; Hsieh et al., 2017). In PD, clinical studies have reported that men are more susceptible to cognitive deficits, while in women there is a lower prevalence of cognitive impairment (Cereda et al., 2016; Reekes et al., 2020).

In our study, we showed a reduction in TH immunoreactivity in the substantia nigra 48 h after the 15th reserpine injection in males. However, female rats presented a lower susceptibility to the deleterious effects of reserpine on DA neurons, which reinforces the evidence regarding the protective effects of estrogen. A possible explanation concerning estrogen effects in females includes the neuroprotective effects on DA neurons (Hyman et al., 1991) as well as the maintenance of neural activity upregulated by BDNF (Cosgrove et al., 2007). Interestingly, a previous report from our group showed that repeated low-dose reserpine administration promotes a reduction in BDNF in the hippocampus of male mice (Campelo et al., 2017). Accordingly, the reduction of BDNF in the blood of PD patients of both sexes was demonstrated Wang et al. (2016) and a meta-analysis reported a more prominent reduction of BDNF in men (Rahmani et al., 2019).

It should be noted that the reserpine-induced reduction in TH could be related to a feedback inhibition of TH expression in DA neurons. In this respect, besides TH decrement in the nigrostriatal pathway, reserpine also causes increase in oxidative stress parameters (Fernandes et al., 2012; Silva-Martins et al., 2021), increase in alpha-synuclein expression (Leão et al., 2017), alterations in axonal ultrastructure (Leal et al., 2019) and increase of neuroinflammation parameters (unpublished data). However, despite the several indicators that repeated reserpine induces some extent of neuronal damage compatible with Parkinson’s disease, it is important to mention that the TH decrement was partially recovered after prolonged withdrawal (in male rats—Santos et al., 2013). Thus, the repeated reserpine model comprises important validations as an animal model of progressive parkinsonism, but the extent of neuronal damage and the presence of neuronal loss are yet to be determined. Regardless, our study shows a protection against behavioral impairment and TH decrement in female subjects, which can be related to estrogen neuroprotective effects such as anti-inflammatory, antioxidant, neurotrophic actions, among others (see Dluzen, 2000 for a review). Alternatively, there are sex differences in the basal physiology of dopaminergic neurotransmission, possibly mediated by estrogen (Watson et al., 2006; Haaxma et al., 2007; McArthur et al., 2007) which can counteract the effect of reserpine on dopamine turnover. Specifically, estradiol may increase dopamine release in the striatum (Becker, 1990; Morissette et al., 2008) and increase the DA turnover by modulating the DA transporter (DAT, McArthur et al., 2007).

Our results are in accordance with the lower incidence of PD in women (Gillies et al., 2014) and reinforces the valuable contribution of this model in evaluating different characteristics associated with PD, especially regarding sex differences in clinical motor and non-motor symptoms of the prodromal phase.

The results related to the estrous cycle evaluation revealed a counter-balanced distribution of the female rats across phases throughout the experimental design (Figure 7). As mentioned, several studies have suggested that sex hormones, mainly estrogen, play an essential role in these sex differences in animal models of PD. In this regard, after the 15th injection of the treatment regimen, we observed that reserpine-treated females present the estradiol levels inversely associated with motor performance in the catalepsy task. In other words, female rats with the lowest estradiol levels showed the highest duration of catalepsy. In contrast, for the vehicle group, we did not observe an association between hormonal levels and motor performance along the experimental protocol. These data corroborate previous studies showing that estrogen treatment (Saunders-Pullman et al., 1999; Tsang et al., 2000; Rajsombath et al., 2019) and prolonged natural exposure to endogenous estrogens (Saunders-Pullman et al., 1999) reduced the symptoms and risk of developing PD. In addition, physiological levels of estrogen, as well as exogenous reposition of this hormone protect females against nigrostriatal injury (Yu and Liao, 2000; Datla et al., 2003; Dluzen and Horstink, 2003). In the MPTP model, ovariectomized females mice showed a higher striatal DA depletion compared with gonadal intact females (Dluzen et al., 1996). In female rats, the replacement of estradiol levels reversed this DA depletion in studies using the 6-OHDA model (Murray et al., 2003), although the physiological mechanisms are not yet elucidated.

As mentioned, there is evidence that estrogens act as a neuroprotective factor for dopaminergic neurons in several animal models of PD, such as induced by 6-OHDA (Tamas et al., 2005) and MPTP (Ookubo et al., 2009; Antzoulatos et al., 2010). However, it has been reported that the effects of these neurotoxins may depend upon the specific stage of the estrous cycle the females are in when injected the toxin (Yu and Liao, 2000). For example, an extended loss of DA neurons occurs in phases of low estrogen levels (Yu and Liao, 2000). In this sense, due to the progressive nature of the PD model used here, our results have shown a protection against reserpine-induced alterations during the early stage in females, when there are still regular estrous cycles.

In summary, we report a sexually dimorphic profile of parkinsonism progression induced by reserpine, consistent with the clinical literature and acute animal models of PD using neurotoxins. We also showed a correlation between low levels of estrogen and increased catalepsy, suggesting a protective role of this hormone. However, further studies are required to elucidate the specific role of sex hormones on the behavior and neurochemical mechanisms related to sexual dimorphism in the development of reserpine-induced parkinsonism.

## Data Availability Statement

The raw data supporting the conclusions of this article will be made available by the authors, without undue reservation.

## Ethics Statement

The animal study was reviewed and approved by the Comissão de Ética no Uso de Animais—UNIFESP.

## Author Contributions

AL conducted the experiments and data analysis. AL, YM, and RS wrote the manuscript. VB, DC, NG, LL-S, MB, MS, and GM participated in experiments, data collection, and data analysis. YM, JS, and RS designed the study. JS contributed in writing. RS coordinated the study. All authors contributed to the article and approved the submitted version.

## Conflict of Interest

The authors declare that the research was conducted in the absence of any commercial or financial relationships that could be construed as a potential conflict of interest.

## Publisher’s Note

All claims expressed in this article are solely those of the authors and do not necessarily represent those of their affiliated organizations, or those of the publisher, the editors and the reviewers. Any product that may be evaluated in this article, or claim that may be made by its manufacturer, is not guaranteed or endorsed by the publisher.
