# Supplementary material for: Female Rats Are Resistant to Cognitive, Motor and Dopaminergic Deficits in the Reserpine-Induced Progressive Model of Parkinson’s Disease
Source: Front Aging Neurosci. 2021 Oct 25;13:757714. doi: 10.3389/fnagi.2021.757714 (PMC8573221; doi:10.3389/fnagi.2021.757714)
Supplement: Supplementary file 1 [file Table_1.docx]

Table 1: Original data of catalepsy test (displayed in figure 2A).

| **#** | **Sex** | **Treatment** | **Time of injections** | **Basal** | **1^st^** | **2^nd^** | **3^rd^** | **4^th^** | **5^th^** | **6^th^** | **7^th^** | **8^th^** | **9^th^** | **10^th^** | **11^th^** | **12^th^** | **13^th^** | **14^th^** | **15^th^** |
| --- | --- | --- | --- | --- | --- | --- | --- | --- | --- | --- | --- | --- | --- | --- | --- | --- | --- | --- | --- |
| 1 | Male | Veh | 10 | 0.34 | 0.33 | 0.47 | 1.63 | 0.53 | 1.92 | 1.94 | 1.25 | 2.71 | 1.23 | 6.39 |  |  |  |  |  |
| 2 |  |  |  | 0.29 | 0.37 | 0.55 | 2.09 | 1.97 | 5.07 | 1.79 | 2.28 | 2.48 | 2.56 | 4.33 |  |  |  |  |  |
| 3 |  |  |  | 0.27 | 0.90 | 0.65 | 1.02 | 1.17 | 3.20 | 2.64 | 2.92 | 2.34 | 4.62 | 4.07 |  |  |  |  |  |
| 4 |  |  |  | 0.27 | 0.39 | 1.47 | 3.02 | 2.55 | 1.82 | 1.36 | 0.96 | 1.08 | 0.58 | 0.69 |  |  |  |  |  |
| 5 |  |  |  | 0.27 | 0.40 | 1.95 | 2.73 | 3.41 | 3.86 | 2.02 | 2.47 | 4.32 | 4.77 | 3.30 |  |  |  |  |  |
| 6 |  |  | 15 | 0.35 | 0.45 | 1.95 | 0.78 | 2.38 | 2.55 | 2.69 | 2.21 | 0.97 | 0.84 | 2.53 | 2.26 | 0.48 | 2.40 | 5.74 | 5.68 |
| 7 |  |  |  | 0.35 | 0.47 | 0.50 | 0.79 | 1.38 | 0.95 | 1.71 | 1.01 | 1.99 | 0.47 | 0.69 | 1.58 | 0.62 | 1.31 | 1.56 | 2.60 |
| 8 |  |  |  | 0.50 | 0.49 | 1.02 | 1.26 | 2.43 | 0.71 | 1.29 | 2.27 | 2.02 | 2.15 | 1.81 | 2.42 | 1.22 | 0.83 | 0.92 | 2.98 |
| 9 |  |  |  | 0.66 | 0.73 | 0.51 | 1.38 | 1.31 | 2.71 | 1.98 | 2.15 | 2.74 | 3.55 | 7.26 | 2.66 | 5.16 | 5.52 | 6.66 | 9.25 |
| 10 |  |  |  | 0.59 | 0.76 | 0.51 | 0.48 | 1.72 | 1.82 | 2.14 | 1.45 | 1.16 | 1.85 | 2.42 | 0.98 | 2.84 | 2.10 | 1.13 | 3.24 |
| 11 |  | Res | 10 | 0.31 | 0.35 | 1.19 | 1.99 | 2.52 | 2.74 | 6.95 | 6.85 | 7.22 | 7.73 | 9.86 |  |  |  |  |  |
| 12 |  |  |  | 0.40 | 0.32 | 0.45 | 0.85 | 0.52 | 2.21 | 1.30 | 1.52 | 6.03 | 6.04 | 10.60 |  |  |  |  |  |
| 13 |  |  |  | 0.32 | 0.75 | 1.44 | 2.19 | 2.33 | 6.16 | 5.88 | 4.29 | 4.27 | 10.00 | 3.92 |  |  |  |  |  |
| 14 |  |  |  | 0.27 | 0.48 | 1.08 | 0.94 | 1.77 | 3.00 | 4.71 | 3.92 | 6.65 | 4.55 | 12.51 |  |  |  |  |  |
| 15 |  |  |  | 0.29 | 0.34 | 0.41 | 0.82 | 1.21 | 2.04 | 3.32 | 1.62 | 2.07 | 4.45 | 7.44 |  |  |  |  |  |
| 16 |  |  | 15 | 0.53 | 0.37 | 0.44 | 1.95 | 1.26 | 1.64 | 2.15 | 3.99 | 4.36 | 10.86 | 13.87 | 15.61 | 26.93 | 27.54 | 21.37 | 38.19 |
| 17 |  |  |  | 0.32 | 0.42 | 0.61 | 0.40 | 0.95 | 2.08 | 3.32 | 3.28 | 4.10 | 7.58 | 12.22 | 17.32 | 46.92 | 46.54 | 29.84 | 35.99 |
| 18 |  |  |  | 0.40 | 0.42 | 0.49 | 0.96 | 1.39 | 4.41 | 4.41 | 3.39 | 4.81 | 6.36 | 7.31 | 7.73 | 7.49 | 9.18 | 16.24 | 13.14 |
| 19 |  |  |  | 1.30 | 0.60 | 0.75 | 1.35 | 2.42 | 2.83 | 6.69 | 2.80 | 3.59 | 6.20 | 3.98 | 7.20 | 10.55 | 19.81 | 37.92 | 82.91 |
| 20 |  |  |  | 0.77 | 1.01 | 1.32 | 0.97 | 0.61 | 7.40 | 6.27 | 8.15 | 8.73 | 13.50 | 20.61 | 47.60 | 34.95 | 27.28 | 38.90 | 35.62 |
| 21 | Female | Veh | 10 | 0.26 | 0.33 | 0.40 | 1.30 | 0.99 | 1.39 | 1.81 | 3.70 | 2.93 | 3.67 | 3.25 |  |  |  |  |  |
| 22 |  |  |  | 0.26 | 0.33 | 0.45 | 1.49 | 1.44 | 1.47 | 2.27 | 1.84 | 1.88 | 1.05 | 2.97 |  |  |  |  |  |
| 23 |  |  |  | 0.27 | 0.33 | 0.38 | 0.51 | 1.56 | 4.80 | 4.82 | 4.03 | 1.03 | 1.33 | 1.94 |  |  |  |  |  |
| 24 |  |  |  | 0.27 | 0.27 | 0.39 | 0.53 | 0.66 | 0.93 | 1.46 | 1.58 | 1.33 | 3.28 | 1.57 |  |  |  |  |  |
| 25 |  |  |  | 0.25 | 0.32 | 0.72 | 1.08 | 2.15 | 0.99 | 2.47 | 1.97 | 1.95 | 1.18 | 2.68 |  |  |  |  |  |
| 26 |  |  |  | 0.36 | 0.40 | 0.64 | 1.34 | 8.85 | 11.02 | 5.25 | 7.12 | 7.20 | 6.89 | 12.30 |  |  |  |  |  |
| 27 |  |  |  | 0.33 | 0.47 | 1.27 | 1.95 | 3.29 | 2.31 | 1.44 | 2.07 | 1.90 | 4.40 | 3.27 |  |  |  |  |  |
| 28 |  |  |  | 0.27 | 0.46 | 0.36 | 0.39 | 0.46 | 0.70 | 0.91 | 1.22 | 0.65 | 0.73 | 1.15 |  |  |  |  |  |
| 29 |  |  |  | 0.51 | 1.09 | 2.17 | 3.37 | 1.54 | 2.23 | 1.90 | 1.51 | 2.12 | 1.74 | 2.47 |  |  |  |  |  |
| 30 |  |  |  | 0.56 | 0.70 | 0.47 | 0.64 | 1.11 | 2.20 | 0.48 | 0.61 | 1.33 | 1.40 | 1.11 |  |  |  |  |  |
| 31 |  |  | 15 | 0.84 | 0.72 | 0.69 | 1.07 | 1.07 | 1.60 | 1.48 | 2.45 | 2.63 | 1.89 | 2.52 | 2.90 | 3.01 | 2.91 | 4.74 | 4.41 |
| 32 |  |  |  | 0.35 | 0.43 | 0.51 | 0.42 | 0.69 | 0.50 | 0.58 | 0.59 | 0.49 | 0.56 | 1.14 | 0.93 | 0.67 | 0.83 | 0.73 | 2.82 |
| 33 |  |  |  | 0.47 | 0.47 | 0.39 | 0.54 | 1.24 | 1.82 | 0.89 | 0.83 | 1.61 | 1.70 | 2.30 | 3.03 | 2.34 | 1.67 | 3.17 | 2.36 |
| 34 |  |  |  | 0.38 | 0.36 | 0.74 | 0.62 | 0.80 | 0.71 | 0.92 | 1.62 | 1.57 | 1.05 | 1.91 | 2.47 | 1.74 | 2.25 | 1.29 | 1.57 |
| 35 |  |  |  | 0.31 | 0.43 | 0.52 | 0.48 | 1.26 | 2.01 | 0.84 | 1.11 | 0.88 | 0.59 | 1.67 | 0.59 | 0.61 | 0.64 | 1.85 | 1.05 |
| 36 |  |  |  | 0.38 | 0.81 | 0.68 | 1.08 | 1.97 | 2.65 | 2.65 | 7.05 | 4.83 | 9.24 | 8.36 | 8.70 | 11.22 | 14.02 | 12.14 | 5.52 |
| 37 |  |  |  | 0.29 | 0.46 | 0.76 | 0.89 | 0.89 | 0.74 | 0.74 | 2.87 | 2.47 | 3.66 | 2.13 | 1.11 | 0.89 | 0.79 | 1.50 | 1.77 |
| 38 |  |  |  | 0.29 | 0.59 | 0.91 | 2.83 | 4.34 | 4.04 | 1.89 | 1.41 | 2.00 | 5.48 | 1.64 | 3.30 | 1.84 | 6.74 | 3.64 | 2.50 |
| 39 |  |  |  | 0.22 | 0.46 | 1.38 | 1.01 | 0.69 | 0.60 | 0.57 | 0.89 | 0.88 | 1.48 | 1.49 | 1.46 | 1.91 | 1.24 | 3.20 | 3.10 |
| 40 |  |  |  | 0.29 | 0.48 | 0.59 | 0.58 | 1.00 | 3.15 | 0.67 | 1.45 | 2.54 | 3.73 | 2.12 | 5.69 | 3.99 | 3.35 | 3.72 | 2.78 |
| 41 |  | Res | 10 | 0.25 | 0.35 | 0.45 | 1.66 | 0.48 | 1.81 | 2.52 | 0.90 | 6.02 | 5.00 | 4.83 |  |  |  |  |  |
| 42 |  |  |  | 0.24 | 0.35 | 0.32 | 0.94 | 0.74 | 2.07 | 2.79 | 7.27 | 4.76 | 5.08 | 10.52 |  |  |  |  |  |
| 43 |  |  |  | 0.26 | 1.01 | 1.54 | 1.75 | 4.88 | 1.94 | 4.39 | 1.41 | 3.13 | 5.40 | 7.69 |  |  |  |  |  |
| 44 |  |  |  | 0.28 | 0.32 | 0.98 | 1.95 | 4.08 | 3.89 | 5.76 | 7.09 | 5.48 | 13.88 | 13.27 |  |  |  |  |  |
| 45 |  |  |  | 0.25 | 0.36 | 0.77 | 1.16 | 1.09 | 0.68 | 2.07 | 0.98 | 0.73 | 0.86 | 1.86 |  |  |  |  |  |
| 46 |  |  |  | 0.37 | 0.52 | 0.92 | 3.91 | 7.27 | 11.16 | 14.01 | 5.43 | 10.57 | 11.43 | 14.49 |  |  |  |  |  |
| 47 |  |  |  | 0.46 | 0.58 | 0.58 | 0.74 | 1.34 | 1.20 | 1.13 | 0.78 | 1.39 | 4.05 | 3.06 |  |  |  |  |  |
| 48 |  |  |  | 0.25 | 0.43 | 0.66 | 1.79 | 1.79 | 1.69 | 1.64 | 2.52 | 1.60 | 8.12 | 5.77 |  |  |  |  |  |
| 49 |  |  |  | 0.37 | 0.46 | 1.41 | 2.15 | 3.05 | 4.32 | 2.70 | 4.32 | 4.31 | 5.34 | 4.68 |  |  |  |  |  |
| 50 |  |  |  | 0.48 | 0.79 | 1.38 | 3.04 | 2.63 | 2.82 | 4.87 | 3.76 | 2.54 | 3.74 | 4.25 |  |  |  |  |  |
| 51 |  |  | 15 | 0.56 | 0.43 | 0.52 | 1.69 | 1.47 | 2.78 | 3.89 | 4.61 | 7.63 | 9.14 | 13.47 | 31.99 | 27.74 | 46.22 | 63.09 | 41.39 |
| 52 |  |  |  | 0.38 | 0.60 | 0.43 | 1.06 | 1.19 | 1.45 | 1.55 | 0.66 | 0.84 | 2.41 | 1.91 | 3.34 | 2.59 | 4.58 | 4.35 | 4.05 |
| 53 |  |  |  | 0.38 | 0.46 | 0.33 | 0.47 | 0.86 | 0.85 | 0.63 | 1.81 | 1.61 | 4.18 | 3.29 | 5.94 | 10.97 | 12.57 | 23.55 | 84.18 |
| 54 |  |  |  | 0.41 | 0.40 | 0.59 | 1.33 | 2.06 | 2.81 | 10.92 | 4.92 | 3.90 | 5.89 | 7.42 | 29.52 | 14.79 | 23.44 | 35.12 | 62.68 |
| 55 |  |  |  | 0.38 | 0.47 | 0.91 | 0.60 | 0.70 | 1.09 | 1.31 | 1.58 | 1.97 | 3.39 | 2.14 | 4.97 | 2.98 | 4.23 | 5.76 | 6.53 |
| 56 |  |  |  | 0.32 | 0.40 | 0.70 | 1.78 | 2.18 | 2.64 | 3.34 | 3.00 | 3.72 | 4.65 | 4.64 | 3.41 | 5.13 | 8.21 | 6.98 | 4.83 |
| 57 |  |  |  | 0.29 | 0.52 | 0.73 | 1.15 | 1.68 | 1.06 | 3.86 | 3.18 | 2.52 | 5.19 | 3.46 | 8.69 | 6.10 | 6.72 | 6.25 | 6.83 |
| 58 |  |  |  | 0.30 | 0.64 | 0.97 | 1.00 | 1.27 | 2.12 | 0.97 | 1.06 | 2.25 | 2.27 | 3.64 | 3.23 | 4.00 | 6.37 | 3.08 | 2.98 |
| 59 |  |  |  | 0.33 | 0.51 | 0.54 | 0.51 | 0.96 | 1.22 | 0.80 | 0.76 | 0.69 | 2.75 | 2.02 | 2.13 | 1.62 | 1.19 | 1.97 | 3.53 |
| 60 |  |  |  | 0.64 | 0.60 | 0.60 | 1.09 | 5.73 | 1.78 | 2.41 | 3.06 | 3.09 | 2.56 | 1.82 | 2.16 | 2.89 | 2.17 | 1.62 | 1.61 |
