# Supplementary material for: Female Rats Are Resistant to Cognitive, Motor and Dopaminergic Deficits in the Reserpine-Induced Progressive Model of Parkinson’s Disease
Source: Front Aging Neurosci. 2021 Oct 25;13:757714. doi: 10.3389/fnagi.2021.757714 (PMC8573221; doi:10.3389/fnagi.2021.757714)
Supplement: Supplementary file 2 [file Table_2.DOCX]

Table 2: Original data of catalepsy test compiled in phases (displayed in figure 2B).

| **#** | **Sex** | **Treatment** | **Time of injections** | **Basal** | **Prodromal phase** | **Early motor phase** | **Late motor phase** |
| --- | --- | --- | --- | --- | --- | --- | --- |
| 1 | Male | Veh | 10 | 0,34 | 0,97 | 2,71 |  |
| 2 |  |  |  | 0,29 | 2,01 | 2,69 |  |
| 3 |  |  |  | 0,27 | 1,39 | 3,32 |  |
| 4 |  |  |  | 0,27 | 1,85 | 0,93 |  |
| 5 |  |  |  | 0,27 | 2,47 | 3,37 |  |
| 6 |  |  | 15 | 0,35 | 1,62 | 1,85 | 3,31 |
| 7 |  |  |  | 0,35 | 0,82 | 1,17 | 1,53 |
| 8 |  |  |  | 0,50 | 1,18 | 1,91 | 1,67 |
| 9 |  |  |  | 0,66 | 1,33 | 3,53 | 5,85 |
| 10 |  |  |  | 0,59 | 1,06 | 1,80 | 2,06 |
| 31 |  | Res | 10 | 0,31 | 1,76 | 7,72 |  |
| 32 |  |  |  | 0,40 | 0,87 | 5,10 |  |
| 33 |  |  |  | 0,32 | 2,57 | 5,67 |  |
| 34 |  |  |  | 0,27 | 1,45 | 6,47 |  |
| 35 |  |  |  | 0,29 | 0,96 | 3,78 |  |
| 36 |  |  | 15 | 0,53 | 1,13 | 7,05 | 25,93 |
| 37 |  |  |  | 0,32 | 0,89 | 6,10 | 35,32 |
| 38 |  |  |  | 0,40 | 1,53 | 5,25 | 10,76 |
| 39 |  |  |  | 1,30 | 1,59 | 4,65 | 31,68 |
| 40 |  |  |  | 0,77 | 2,26 | 11,45 | 36,87 |
| 11 | Female | Veh | 10 | 0,26 | 0,88 | 3,07 |  |
| 12 |  |  |  | 0,26 | 1,04 | 2,00 |  |
| 13 |  |  |  | 0,27 | 1,51 | 2,63 |  |
| 14 |  |  |  | 0,27 | 0,55 | 1,84 |  |
| 15 |  |  |  | 0,25 | 1,05 | 2,05 |  |
| 16 |  |  |  | 0,36 | 4,45 | 7,75 |  |
| 17 |  |  |  | 0,33 | 1,86 | 2,61 |  |
| 18 |  |  |  | 0,27 | 0,48 | 0,93 |  |
| 19 |  |  |  | 0,51 | 2,08 | 1,95 |  |
| 20 |  |  |  | 0,56 | 1,02 | 0,99 |  |
| 21 |  |  | 15 | 0,84 | 1,03 | 2,19 | 3,59 |
| 22 |  |  |  | 0,35 | 0,51 | 0,67 | 1,20 |
| 23 |  |  |  | 0,47 | 0,89 | 1,47 | 2,51 |
| 24 |  |  |  | 0,38 | 0,65 | 1,42 | 1,86 |
| 25 |  |  |  | 0,31 | 0,94 | 1,02 | 0,95 |
| 26 |  |  |  | 0,38 | 1,44 | 6,43 | 10,32 |
| 27 |  |  |  | 0,29 | 0,75 | 2,37 | 1,21 |
| 28 |  |  |  | 0,29 | 2,54 | 2,48 | 3,60 |
| 29 |  |  |  | 0,22 | 0,83 | 1,06 | 2,18 |
| 30 |  |  |  | 0,29 | 1,16 | 2,10 | 3,91 |
| 41 |  | Res | 10 | 0,25 | 0,95 | 3,85 |  |
| 42 |  |  |  | 0,24 | 0,88 | 6,09 |  |
| 43 |  |  |  | 0,26 | 2,22 | 4,40 |  |
| 44 |  |  |  | 0,28 | 2,24 | 9,10 |  |
| 45 |  |  |  | 0,25 | 0,81 | 1,30 |  |
| 46 |  |  |  | 0,37 | 4,76 | 11,19 |  |
| 47 |  |  |  | 0,46 | 0,89 | 2,08 |  |
| 48 |  |  |  | 0,25 | 1,27 | 3,93 |  |
| 49 |  |  |  | 0,37 | 2,28 | 4,27 |  |
| 50 |  |  |  | 0,48 | 2,13 | 3,83 |  |
| 51 |  |  | 15 | 0,56 | 1,38 | 7,75 | 42,09 |
| 52 |  |  |  | 0,38 | 0,95 | 1,47 | 3,78 |
| 53 |  |  |  | 0,38 | 0,59 | 2,30 | 27,44 |
| 54 |  |  |  | 0,41 | 1,44 | 6,61 | 33,11 |
| 55 |  |  |  | 0,38 | 0,75 | 2,08 | 4,89 |
| 56 |  |  |  | 0,32 | 1,54 | 3,87 | 5,71 |
| 57 |  |  |  | 0,29 | 1,03 | 3,64 | 6,92 |
| 58 |  |  |  | 0,30 | 1,20 | 2,04 | 3,93 |
| 59 |  |  |  | 0,33 | 0,74 | 1,40 | 2,09 |
| 60 |  |  |  | 0,64 | 1,96 | 2,59 | 2,09 |
