# Supplementary material for: Female Rats Are Resistant to Cognitive, Motor and Dopaminergic Deficits in the Reserpine-Induced Progressive Model of Parkinson’s Disease
Source: Front Aging Neurosci. 2021 Oct 25;13:757714. doi: 10.3389/fnagi.2021.757714 (PMC8573221; doi:10.3389/fnagi.2021.757714)
Supplement: Supplementary file 3 [file Table_3.docx]

Table 3: Original data of TH+ neurons, displayed in figure 6B.

|  |  |  |  | **Counting immunoreactive TH neurons in SNPc area** | | | | | | | | | | | | |
| --- | --- | --- | --- | --- | --- | --- | --- | --- | --- | --- | --- | --- | --- | --- | --- | --- |
| **#** | **Treatment** | **Sex** | **Number of injections** | **Section 1** | **Section 2** | **Section 3** | **Section 4** | **Section 5** | **Section 6** | **Section 7** | **Section 8** | **Section 9** | **Section 10** | **Section 11** | **Section 12** |  |
| 1 | Veh | Male | 10 | 53 | 88 | 96 | 40 | 67 | 70 | 40 | 64 | 37 | 63 | 66 | 44 |  |
| 2 |  |  |  | 63 | 54 | 61 | 62 | 67 | 64 | 48 | 59 | 66 | 45 | 73 | 68 |  |
| 3 |  |  |  | 66 | 35 | 72 | 66 | 63 | 49 | 59 | 52 | 56 | 48 | 41 | 90 |  |
| 4 |  |  |  | 59 | 91 | 60 | 59 | 55 | 66 | 56 | 49 | 37 | 87 | 87 | 63 |  |
| 5 |  |  |  | 85 | 90 | 88 | 91 | 41 | 58 | 53 | 40 | 67 | 54 | 54 | 82 |  |
| 6 |  |  | 15 | 58 | 63 | 54 | 65 | 63 | 58 | 65 | 52 | 63 | 60 | 79 | 43 |  |
| 7 |  |  |  | 59 | 75 | 76 | 63 | 78 | 76 | 92 | 72 | 100 | 81 | 74 | N/A |  |
| 8 |  |  |  | 38 | 76 | 75 | 69 | 71 | 72 | 81 | 49 | 43 | 58 | 38 | N/A |  |
| 9 |  |  |  | 32 | 74 | 24 | 56 | 45 | 63 | 22 | 52 | 108 | 92 | 97 | 80 |  |
| 10 |  |  |  | 65 | 83 | 87 | 71 | 59 | 65 | 78 | 63 | 55 | 67 | 49 | 83 |  |
| 11 |  | Female | 10 | 83 | 60 | 45 | 38 | 75 | 26 | 65 | 39 | 52 | 34 | 75 | 53 |  |
| 12 |  |  |  | 27 | 35 | 56 | 65 | 43 | 58 | 27 | 70 | 40 | 67 | 41 | 43 |  |
| 13 |  |  |  | 35 | 61 | 52 | 75 | 51 | 95 | 73 | 95 | 56 | 108 | 67 | 35 |  |
| 14 |  |  |  | 22 | 9 | 35 |  | 62 | 46 | 53 | 60 | 58 | 65 | 65 | 63 |  |
| 15 |  |  |  | 66 | 101 | 110 | 97 | 81 | 59 | 114 | 31 | 82 | 116 | 88 | N/A |  |
| 16 |  |  | 15 | 31 | 32 | 84 | 94 | 83 | 71 | 66 | 81 | 109 | 102 | 118 | 111 |  |
| 17 |  |  |  | 15 | 45 | 41 | 57 | 32 | 58 | 50 | 68 | 78 | 70 | 53 | 63 |  |
| 18 |  |  |  | N/A | N/A | N/A | N/A | N/A | N/A | N/A | N/A | N/A | N/A | N/A | N/A |  |
| 19 |  |  |  | 70 | 86 | 75 | 87 | 70 | 66 | 112 | 64 | 74 | 98 | N/A | N/A |  |
| 20 |  |  |  | 51 | 59 | 53 | 73 | 77 | 84 | 69 | 77 | N/A | N/A | N/A | N/A |  |
| 21 | Res | Male | 10 | 57 | 91 | 88 | 82 | 51 | 66 | 92 | 81 | 74 | 64 | 46 | 82 |  |
| 22 |  |  |  | 70 | 61 | 47 | 75 | 52 | 53 | 48 | 43 | 40 | 44 | 44 | 67 |  |
| 23 |  |  |  | 45 | 69 | 62 | 72 | 74 | 64 | 82 | 86 | 60 | 55 | 61 | 65 |  |
| 24 |  |  |  | 35 | 8 | 48 | 37 | 47 | 47 | 63 | 54 | 55 | 42 | 61 | 82 |  |
| 25 |  |  |  | N/A | N/A | N/A | N/A | N/A | N/A | N/A | N/A | N/A | N/A | N/A | N/A |  |
| 26 |  |  | 15 | 62 | 34 | 56 | 28 | 39 | 28 | 36 | 47 | 68 | 95 | 64 | N/A |  |
| 27 |  |  |  | 76 | 37 | 72 | 64 | 63 | 48 | 59 | 53 | 52 | 58 | 47 | 80 |  |
| 28 |  |  |  | 35 | 53 | 65 | 43 | 66 | 52 | 58 | 78 | 68 | 72 | 58 | 68 |  |
| 29 |  |  |  | 73 | 64 | 67 | 54 | 44 | 33 | 56 | 49 | 66 | 60 | N/A | N/A |  |
| 30 |  |  |  | 59 | 43 | 41 | 54 | 21 | 38 | 30 | 59 | 30 | 60 | 58 | 63 |  |
| 31 |  | Female | 10 | 35 | 8 | 48 | 37 | 47 | 47 | 63 | 54 | 55 | 42 | 61 | 82 |  |
| 32 |  |  |  | 34 | 5 | 75 | 67 | 56 | 51 | 49 | 75 | 61 | 60 | 49 | 71 |  |
| 33 |  |  |  | 7 | 63 | 51 | 54 | 30 | 66 | 57 | 61 | 62 | 84 | 54 | 91 |  |
| 34 |  |  |  | 53 | 71 | 52 | 48 | 64 | 55 | 64 | 71 | 54 | 95 | 62 | 82 |  |
| 35 |  |  |  | 42 | 100 | 52 | 74 | 6 | 91 | 24 | 91 | 32 | 76 | 79 | 85 |  |
| 36 |  |  | 15 | 48 | 59 | 56 | 34 | 80 | 43 | 67 | 46 | 103 | 52 | 91 | 111 |  |
| 37 |  |  |  | 79 | 49 | 79 | 35 | 56 | 60 | 73 | 49 | 70 | 47 | N/A | N/A |  |
| 38 |  |  |  | 41 | 55 | 68 | 24 | 67 | 29 | 73 | 41 | 49 | 29 | 69 | 33 |  |
| 39 |  |  |  | 90 | 79 | 52 | 50 | 73 | 60 | 103 | 77 | 80 | 79 | 58 | 64 |  |
| 40 |  |  |  | 62 | 8 | 74 | 119 | 59 | 49 | 73 | 47 | 55 | 31 | 73 | 76 |  |
|  | **N/A: not available** | |  |  |  |  |  |  |  |  |  |  |  |  |  |  |
